# Health and health management among motorcycle-based food delivery workers in South Korea: a qualitative interview study

**DOI:** 10.1080/17482631.2026.2613971

**Published:** 2026-01-11

**Authors:** Sookyung Kim, Min Soo Woo, Soyun Hong

**Affiliations:** a School of Nursing, College of Medicine, Soonchunhyang University, Cheonan, Republic of Korea; b Department of Nursing, Keimyung College University, Daegu, Republic of Korea; c Department of Nursing, Korean Bible University, Seoul, Republic of Korea

**Keywords:** Qualitative research, platform workers, occupational health, motorcycle-based food delivery workers, Health management

## Abstract

**Purpose:**

This study aimed to qualitatively examine the daily lives of motorcycle-based food delivery workers, focusing on how they experience, perceive, and interpret their health-related issues.

**Methods:**

Semi-structured in-depth interviews were conducted with nine MFDWs in South Korea between July and September 2024 to explore their perceptions of health. Participants were recruited through purposive and snowball sampling, and data were analyzed using thematic analysis.

**Results:**

Thematic analysis revealed the following key findings: MFDWs' challenging working conditions posed physical and emotional stressors, which contributed to negligent driving and unhealthy habits. Although they recognized traffic accidents as the most critical health risk, they exhibited a tendency toward risky driving behaviors. Unhealthy lifestyles were linked to further health deterioration. While the majority showed a passive attitude toward health management, a few adopted individual strategies to maintain their health.

**Conclusions:**

The findings suggest the need for policy-level attention to mitigate traffic accident risk factors among MFDWs. Larger and more diverse studies are required to confirm these findings and to provide a stronger evidence base for policy recommendations. In addition, delivery applications could be further refined to help reduce occupational risks, and the development of tailored health promotion interventions may support their health and well-being.

## Background

The gig and platform economy is rapidly growing owing to advances in information and communication technology, accelerated urbanisation, and a rising demand for contactless services since the COVID-19 pandemic (Acs et al., 2021; European Agency for Safety and Health at Work [EU-OSHA], 2022; European Commission, 2021; Goel et al., 2024). The number of digital labour platforms increased from 142 in 2010 to over 777 in 2020, with taxi and delivery services expanding nearly tenfold (International Labor Organization [ILO], 2021). Among these, motorcycle-based food delivery workers (MFDWs) have emerged as a key segment in response to evolving consumer demands, enabling fast and efficient services through digital platforms (Acs et al., 2021; ILO, 2021). This expansion has transformed the working environment and employment structures, highlighting the urgent need for greater attention and tailored support for this emerging occupational group (Acs et al., 2021; Goel et al., 2024; Ministry of Employment and Labour, 2021).

The MFDWs’ work conditions differ markedly from those found in conventional work environment settings (Acs et al., 2021; Antonucci, 2025; ILO, 2021; Ministry of Employment and Labour, 2021). Traditional jobs involve clear employment contracts, predictable working hours, and systematic provisions for welfare and rest (Acs et al., 2021; Antonucci, 2025; Yu et al., 2024). By contrast, the delivery industry exhibits significant job instability resulting from algorithm-based order distribution, irregular work patterns, extended shifts, and insufficient rest (Employment and Social Development Canada, 2024; Ministry of Business, 2018). MFDWs experience considerably greater economic insecurity and heightened employment uncertainty under unstable conditions than workers in traditional environments (Hajiheydari & Delgosha, 2024; Nguyen et al., 2024; Popan, 2024). Overall, to maintain the occupational health of MFDWs, they must understand their work environment and the nature of their tasks (ILO, 2021; Ministry of Employment and Labour, 2021).

To meet the demand for swift meal deliveries, MFDWs navigate dense urban traffic at high speeds and adhere to strict schedules, exposing themselves to various occupational health and safety risks (Acs et al., 2021; Goel et al., 2024; ILO, 2021). Beyond persistent-road dangers, their customer interactions may expose them to violence, harassment, and other criminal acts. Additionally, extreme weather and chronic congestion exacerbate their psychological strain (Antonucci, 2025; EU-OSHA, 2022; Nilsen & Kongsvik, 2023). A 2021 nationwide survey of 5,626 MFDWs across six major platforms reflected these risks, with 47% reporting at least one delivery-related accident, averaging 2.4 incidents per rider, and 14.9% reported five or more such incidents (Ministry of Employment and Labour, 2021). These findings highlight the importance of integrated occupational health strategies and policy interventions to safeguard MFDWs’ well-being (EU-OSHA, 2022; Ministry of Employment and Labour, 2021; Nilsen & Kongsvik, 2023).

Previous studies on MFDWs’ health have predominantly used quantitative methods to measure the extent of their physical strain, documenting high rates of musculoskeletal pain, chronic fatigue, sleep disturbances, and irregular eating patterns, as well as extended and irregular work hours driven by heavy delivery quotas (Hajiheydari & Delgosha, 2024; Ministry of Employment and Labour, 2021; Nguyen et al., 2024; Popan, 2024). Although these studies offer valuable data on physical strain and work intensity, they provide a limited understanding of the subjective meanings, coping strategies, and contexts that shape riders’ daily experiences and risk-taking behaviours (ILO, 2021; Ministry of Employment and Labour, 2021). Qualitative research is essential to understand the daily lives of specific populations (Creswell & Poth, 2016). Therefore, this study aimed to qualitatively explore the daily lives of MFDWs and to understand how they experience and make sense of their health issues.

## Methods

### Study design

This study used semi-structured in-depth interviews and thematic analysis to explore MFDWs’ perceptions of health status and management. This study was approved by the Institutional Review Board of Soonchunhyang University (IRB No. 202406-SB-065).

### Participants recruitment

Participants were selected through purposive and snowball sampling. Purposive sampling was used to recruit adult MFDWs who had been working full-time for at least six months to obtain rich and relevant data. In addition, snowball sampling was applied by asking participants to introduce other useful informants who could provide in-depth insights into the health experiences of MFDWs. Snowball sampling is particularly useful when researchers cannot easily identify potential participants or when useful informants are not readily accessible (Holloway & Galvin, 2017). The exclusion criterion was holding any other full-time job besides motorcycle-based food delivery. Participants were recruited with the cooperation of food delivery agency managers in regions A and B in South Korea. The managers posted the study announcement on internal bulletin boards or uploaded it to group chat rooms on social networks to aid participant recruitment. MFDWs who were interested in participating directly contacted the researchers using the contact information provided in the notice. The researchers then confirmed eligibility, explained the study purpose, interview procedures, and voluntary participation, and scheduled interviews with those who expressed willingness to participate. Before the in-depth interviews, the researcher explained the study’s purpose and process. Additionally, they were informed that the interviews would be recorded. Participants’ written informed consent was obtained, and each was provided with a copy. As compensation for their participation, they received a 50,000 KRW (approximately USD 35) gift card.

### Data collection

Data were collected between July and September 2024 in South Korea. Before the interviews, the participants completed a brief survey comprising eight questions regarding their gender, age, duration of experience as an MFDW, weekly working hours, daily break hours, and daily number of deliveries. The researcher asked each question aloud to the participants, and their responses were written on paper. Following the survey, in-depth interviews were conducted using a semi-structured guide (Appendix 1) in a quiet and comfortable setting with an average duration of 69 minutes. The main questions focused on the participants’ experiences and challenges related to their health as MFDWs, which involved the following aspects: (a) overall health status; (b) health behaviours, including dietary habits, exercise, alcohol consumption, and smoking; (c) discomfort or difficulties caused by external physical factors; (d) work-related emotional distress; and (e) coping strategies for work-related stress. All interviews were audio-recorded using either a voice recorder or a mobile phone and were subsequently transcribed for analysis.

Before the interviews began, participants were informed of the study’s purpose and objectives. The interviews were conducted by the first author (SK), a female assistant professor in her 30 s with MPH and PhD degrees in nursing, and by the second author (MW), a female doctoral candidate in her 40 s with professional experience in occupational health practice. Both interviewers had prior experience in qualitative research and had no previous relationship with any of the participants.

A total of nine participants were included to achieve data saturation. Data collection and analysis were conducted concurrently. Initial coding and thematic analysis began after the first five interview transcripts were completed, allowing ongoing refinement of codes. It was determined that data saturation was reached after the eighth interview, when no new codes or themes emerged, and subsequent interviews only confirmed the previously identified patterns. One additional interview was conducted to ensure that thematic saturation had indeed been achieved.

### Data analysis

This study used thematic analysis of the in-depth interview data (Braun & Clarke, 2006). The audio recordings were initially transcribed using an automated software, CLOVA Note, version 2.4 (Naver Cloud Corporation, Seongnam, South Korea), and the second author, MW, reviewed and corrected any inaccuracies to complete the final transcripts. The transcriptions were re-read to ensure data familiarity, and meaningful data units were inductively coded. One researcher (MW) conducted the initial coding and first-level analysis. Coding and categorization were systematically managed using Microsoft Excel. The development of themes and subthemes was discussed regularly with another researcher (SK) to ensure analytic rigour and interpretive consistency. Codes were then reviewed and grouped into categories based on conceptual similarity, with each category labelled to represent its underlying meaning. To identify overarching themes, codes with similar contextual meanings were organised while distinguishing conceptually distinct ones. Throughout this iterative process, data were continuously compared, reclassified, and restructured, and the candidate themes and subthemes were refined. The final themes were reviewed and agreed upon by all three researchers. When discrepancies in coding or category labelling occurred, they were resolved through in-depth discussions until full consensus was reached. Participants did not review the summarised findings; however, analytical rigour was ensured through ongoing discussions among the research team members. The credibility of each theme was assessed to ensure it accurately reflected the participants’ underlying meanings and to identify any potential inconsistencies or alternative interpretations. Ultimately, MFDWs’ health status and management were classified into four themes and eight subthemes.

## Results

The participants’ sociodemographic and work characteristics are presented in Table I. The following four main themes were identified for MFDWs’ health status and management: (i) challenging working conditions, (ii) negligent driving, (iii) unhealthy habits, and (iv) passive health coping. These themes were divided into eight subthemes (Table II).

**Table I.** Sociodemographic and work characteristics of study participants (*N* = 9).

No.	Gender	Age	Months of experience as a MFDW	Working hours per week	Daily break hours	Daily delivery numbers
1	M	37	44	72	1.5	50-60
2	M	30	14	66	1	40-60
3	M	47	87	80	1	40-60
4	M	32	15	84	1	42-50
5	M	48	6	72	1–2	50-60
6	M	59	6	48	2	30-35
7	M	42	51	80	3	30-40
8	M	29	72	84	2	30-40
9	M	57	156	84	4–5	30-50

Note: MFDW = motorcycle-based food delivery worker.

**Table II.** Themes and subthemes from in-depth interviews with motorcycle-based food delivery workers.

Themes	Subthemes
Challenging working conditions	Poor physical environment
	Stress-inducing job characteristics
Negligent driving	Risky driving behaviours while working
	Lack of awareness and practice of traffic accident prevention
Unhealthy habits	Unhealthy lifestyle
	Health issues stemming from their delivery work
Passive health coping	Passive attitude toward health management
	Attempting to improve health through personal strategies

### Theme 1: challenging working conditions

Participants emphasised that working conditions significantly influenced the health of MFDWs. They identified physical environmental factors, such as weather conditions and high stress from various interpersonal interactions, that contributed to their overall stress levels. Most notably, they highlighted the intense time pressure stemming from their income being directly dependent on the number of deliveries completed. This theme comprised two subthemes: *poor physical environment,* and *stress-inducing job characteristics.*

#### Subtheme 1: poor physical environment

Participants described that adverse weather conditions influenced their work environment, as it is slippery and difficult to work during rain or snow. They recognised that using motorcycles inherently involves a high risk of accidents. A participant recounted an accident at night as he failed to notice a pothole owing to poor road conditions, causing his phone to fall from his hands. Additionally, the participants complained about the lack of a rest area while waiting for delivery calls, especially when they feel fatigued or the weather is too hot. A participant (MH5) explained, “Summer is brutal. During the monsoon season, being soaked in rain was at least bearable; however, once it ended, the heat became suffocating. This is absolutely miserable, and on top of that, we have to stay fully covered.”

Another participant (MH9) added that, “When accidents occur, if you look at most riders and take off their gear, their arms and legs will be wrecked. Delivery workers often experience accidents.” Furthermore, MH2 said that “Riders require appropriate resting places. I think this is the most essential thing.”

#### Subtheme 2: stress-inducing job characteristics

Participants expressed experiencing high stress levels due to communication with store owners and customers. They emphasised the intense pressure to meet delivery deadlines and maximise the number of deliveries, as their income depends on delivery volume. However, they highlighted that their earnings are inconsistent. Some also observed an overall decline in work volume after the COVID-19 pandemic. Additionally, riders reported being unfairly contracted, while delivery agency managers expressed challenges in workforce management due to low entry barriers for deliverers and high turnover rates. One participant described feeling frustrated by unfair treatment from customers:

*Sometimes I get so frustrated with customers. They eat all the food and then claim it never arrived. Some even take a photo saying there was an insect inside, even when that’s impossible. And sometimes, customers just completely ignore me—as if I’m not even a person. (MH9)*.

Another participant expressed constant anxiety about unstable income and the need to meet higher delivery counts:

*Even with this motorcycle job, I require a steady workflow. I cannot just rush it. My income increases over time, but because it is not much, I start to feel anxious. I keep thinking, ‘I have done 10 deliveries, but I need to hit 20, then 30.’ This pressure continues to accumulate. (MH6)*.

### Theme 2: negligent driving

Most participants recognised motorcycle traffic accidents as the most critical health risk for MFDWs and expressed that avoiding such accidents was a top priority for health maintenance. One participant shared that “Working in this job itself without getting injured seems truly remarkable, as most riders get injured at least once.” A prevalent theme among participants related to traffic accidents was negligent driving during food delivery. This theme comprised two subthemes: *risky driving behaviours while working* and *lack of awareness and practice in traffic accident prevention.*

#### Subtheme 1: risky driving behaviours while working

Participants discussed the causes of negligent driving, highlighting work-related factors, such as the need to accept delivery requests via smartphones while driving. Some participants explained that they felt anxious when they noticed the food was cooling down, and in an attempt to deliver it promptly, they reported increasing their speed or weaving between cars, which sometimes resulted in traffic accidents. A participant stated:

*I frequently check my phone, and sometimes, when I glance at it, I realise that the car in front has already stopped. By the time I hit the brakes, I feel like it is already too late. That is, when I had an accident once. (MH7)*.

*The traffic light was red, but riders always try to move up to the very front, no matter what. They want to go fast. While squeezing between cars, I once hit a car with my delivery box. (MH4)*.

#### Subtheme 2: lack of awareness and practice of traffic accident prevention

Participants mentioned that traffic accidents were more likely to occur when they were not fully focused on riding and were simultaneously engaged in other activities, such as smoking or checking their phone. One participant observed that some riders adopted dangerous postures, such as sitting cross-legged while riding, highlighting their lack of awareness regarding accident prevention.

*Accidents tend to occur when individuals are not focused on riding, such as failing to notice a car approaching from the side or looking straight ahead. It often occurs when people are distracted—smoking with one hand, losing focus, or checking their phone. (MH5)*.

*The upright posture should be like horseback riding that way, it’s easier to react quickly to any situation. But when riders get tired, they tend to lean back against the delivery box. I see young riders sometimes sitting cross-legged or slouching while they ride, and I can’t help but think, ‘Aren’t they going to get into an accident?’ It’s terrifying that split second is all it takes.(MH6)*.

### Theme 3: *unhealthy habits*

Participants developed various unhealthy habits depending on the job characteristics of the MFDWs. They recognised that certain health problems had newly emerged or worsened as a result of their work. This theme comprised two subthemes: *unhealthy lifestyle* and *health issues stemming from their delivery work.*

#### Subtheme 1: unhealthy lifestyle

Major unhealthy habits included irregular eating patterns such as delayed or skipped meals due to delivery times overlapping with mealtimes, disrupted sleep patterns characterised by late sleeping and waking times, heavy smoking, and excessive alcohol consumption or binge drinking.

MH8 described that “With delivery, if there are many orders, you are constantly racing against time. This means that you end up eating late. This is how the system operates.” Another participant explained:

*I rarely eat meals regularly. Most of the time, I either skip meals or eat at odd hours. My eating schedule is all over the place. If I eat a lot, I might have two meals, but often it is just one. (MH3)*.

Similarly, MH6 explained, “I live as a night owl. When I reach home, I cannot simply eat or go straight to sleep. Therefore, I end up watching TV until around 2 or 3 a.m. before finally going to bed.” Another participant described:

*Compared to before I started this job, I smoke a lot more now. During my work hours, I consume almost two packs. After my shift, I do not smoke as much, but while working, I smoke a lot, even while waiting at the red lights, and there are plenty of them. (MH4)*.

#### Subtheme 2: health issues stemming from their delivery work

Participants emphasised the physical issues caused by riding motorcycles during work, including wrist and back pain from prolonged driving, shoulder and neck discomfort due to the helmet’s weight, and increased eye fatigue due to frequent smartphone use to accept delivery requests. Furthermore, they experienced discomfort from foreign particles entering their eyes while driving outdoors because they cannot always keep their helmets closed. Psychological issues were also frequently mentioned, such as sleep disorders and stress caused by time pressure. A participant said:

*Most delivery workers experience back-related problems. Riding a motorcycle is not like sitting comfortably. This is more of a standing position, and its impact is rough. There is constant vibration, and the roads are not perfectly smooth. Every time they hit a bump, the shock is strong. (MH3).*

Another participant explained:

*They always feel something stuck in their eyes. This is uncomfortable and irritating. They are constantly exposed to the wind and dust. Most of them do not usually keep the visor on their helmets down due to the hot weather. Thus, many foreign particles enter, and sometimes bugs fly into their eyes. (MH3).*

Similarly, the participants further stated:

*The helmet itself is heavy, and it’s a real issue. Also, we always keep our phones at an angle on the mounts, the kind of posture where you have to bend down to look at it while riding. Because of that, our necks naturally stick out like turtles, and we get terrible shoulder and neck pain. (MH4).*

*As this job requires us to look at our phones for 12 hours a day, eye strain is inevitable. We must keep checking the screen, not just when accepting orders, but even after that, because we are always preparing for the next one. (MH7).*

*Sometimes, I just cannot fall asleep; even when I am exhausted, my eyes stay wide open. My sleep duration has shortened. Even when I fall asleep, I wake up multiple times at night. Sometimes, I go an entire week, constantly waking up and falling back asleep. At other times, I am unable to sleep until 3 or 4 in the morning. (MH8)*.

*I feel like I have become more impatient since I began this job. I think I was always a little like that, but now I feel like I am constantly racing against time. I have noticed that about myself lately. (MH2)*.

### Theme 4: passive health coping

Participants described various ways in which they managed their health, revealing a predominant tendency to neglect proactive health maintenance. Many expressed that health management was a low priority owing to demanding work schedules and financial constraints, leading to a passive approach to well-being. Some participants reported avoiding medical visits or relying on self-medication. However, a minority of participants actively sought personal strategies to maintain their health within their occupation’s limitations. This theme comprises two subthemes: *passive attitude toward health management* and *attempting to improve health through personal strategies.*

#### Subtheme 1: passive attitude toward health management

Many participants exhibited a passive approach to health management, often prioritising work over personal well-being. They noted that, unlike regular employees receiving periodic health checkups, MFDWs must seek medical care independently, which many find burdensome because of financial constraints and lost income from time off. Some participants were admitted for self-medication rather than visiting a hospital, while others completely disregarded lifestyle modifications, such as dieting, smoking cessation, or reducing alcohol consumption. A participant noted:

*We do not really attend health checkups or anything like this. In regular companies, employees receive periodic checkups, but this is not the case for us. If we feel unwell, we have to go on our own, but honestly, most of us do not really take the initiative to do so. (MH1).*

*Similarly, MH4 said, I rarely visit the hospital. Honestly, it is mainly because of money. if I take a day off, it is not just that I will not make money, I will actually lose it.” Furthermore, MH6 stated, “I just take medicine every day; I do not diet at all, and I have not thought about quitting smoking or drinking”.*

#### Subtheme 2: attempting to improve health through personal strategies

Despite these challenges, some participants attempted to manage their health using individual strategies. Their approaches included adjusting work schedules to reduce fatigue, maintaining hydration, and making minor adjustments to reduce discomfort during work. Some participants emphasised setting personal goals to balance work demands with physical and mental well-being. Although these efforts were limited, they demonstrated a degree of self-initiative in managing their health. MH7 said, “I take my helmet off all the time. Of course, part of the reason is discomfort, such as flattening hair or excessive sweating. Even minor inconveniences such as these can cause strain.” Additionally, MH4 explained, “It is so hot that it feels like my skin is burning. I end up drinking two to three liters of water per day, as this is essentially the only way to cope.” Similarly, MH5 described, “Working long hours really wears me out. I have a target, so even though I normally finish at 11 pm, yesterday, I wrapped up by 9 pm and headed home. Even today, I plan to clock out as soon as I achieve my goal.”

**Figure 1.**
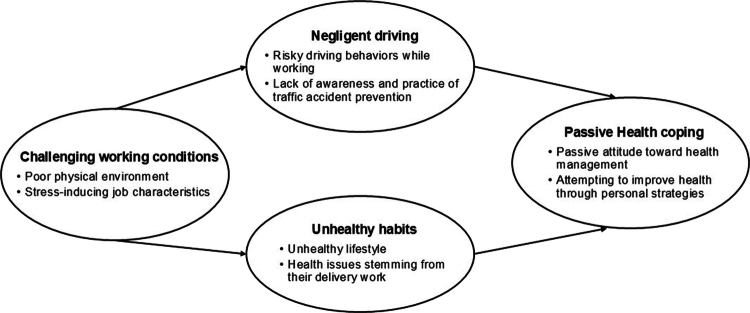
Thamatic map regarding health and health management of motorcycle-based food delivery workers.

Overall, the four themes were interrelated rather than independent (Figure 1). Challenging working conditions characterised by unstable income, time pressure, and exposure to physical and psychological stressors triggered risky driving behaviours and the adoption of unhealthy lifestyles as adaptive responses to job demands. These, in turn, contributed to the deterioration of workers’ health and led to predominantly passive coping strategies toward health management. This interrelated pattern highlights how the structural precarity of platform-based delivery work shapes behavioural, physical, and psychological health outcomes among MFDWs.

## Discussion

This study confirms that negligent driving among MFDWs is a significant cause of traffic accidents. The results revealed that checking mobile phones while driving for food delivery disrupted their observations, significantly reduced driving concentration, and increased the risk of traffic accidents. These findings align with previous research, indicating that the ubiquitous use of delivery applications and the resulting persistent distractions pose a serious threat to driving safety, significantly increasing the risk of traffic accidents (Hajiheydari & Delgosha, 2024; Lee & Park, 2022; Nguyen et al., 2024; Popan, 2024). This underscores the urgent need for technological and institutional improvements to ensure safe delivery services (Nguyen et al., 2024; Popan, 2024). EU-OSHA (2022) has emphasised that digital platform application interfaces should be simplified to enhance more user intuitiveness. Accordingly, delivery platforms should enhance their user interfaces to minimise distractions while driving and implement systems that reduce redundant screen interactions during order confirmation and customer support processes (EU-OSHA, 2022). Such technological and institutional improvements are expected to contribute significantly to ensuring the delivery workers’ safety and reducing the incidence of traffic accidents (Moon and Kim, 2023; Sun, 2023; Vignola et al., 2023; Yu et al., 2024).

This study demonstrates that MFDWs experience significantly poorer working conditions than traditional employees. Participants found that linking income directly to the number of deliveries creates severe time pressure. This finding aligns with a study conducted across multiple countries that reported that MFDWs worked longer hours at a higher intensity to maximise their income, ultimately at the expense of their health (Cheng et al., 2024; Mbare, 2023; Papakostopoulos & Nathanael, 2021; Taylor et al., 2023). Besides this financial strain, unpredictable delivery requests, unfair contractual practices, and high turnover substantially increase work-related stress (Nguyen-Phuoc et al., 2023; Papakostopoulos & Nathanael, 2021). Furthermore, consistent with previous research, this study confirms that adverse weather conditions, such as rain, snow, or extreme heat, combined with unstable road conditions, create a physically challenging environment, further increasing the risk of traffic accidents (Moon et al., 2022; Popan, 2024; Sun, 2023; Vignola et al., 2023). The ILO (2021) report recommends implementing tailored safety training programs that enable workers to quickly identify hazards and develop effective risk management skills. It also advocates for regular, structured breaks and the provision of personal protective equipment appropriate to the job’s specific risk. Effective implementation of these recommendations can significantly reduce both physical and mental fatigue experienced by MFDWs and mitigate direct exposure to occupational hazards (Cheng et al., 2024; ILO, 2021; Nguyen-Phuoc et al., 2023).

MFDWs engage in unhealthy behaviours, such as irregular eating, disrupted sleep, increased smoking, and excessive alcohol consumption. These habits contribute to chronic pain, severe eye strain, and ongoing sleep disturbances, which undermine their overall well-being. Compared with findings from previous studies on MFDWs in other countries, these tendencies appear more pronounced among Korean MFDWs. They work an average of 74 hours per week (Boniardi et al., 2024; Xu & Liu, 2021). Addressing these health issues requires more than simply applying working time regulations, as such measures may reduce income. Therefore, occupational health professionals and researchers should collaborate to develop effective solutions (Papakostopoulos & Nathanael, 2021; Taylor et al., 2023). Several countries have recognised these issues and implemented policies to improve delivery workers’ health (Nguyen-Phuoc et al., 2023; Taylor et al., 2023). For example, the European Union has tightened working hour regulations and mandated rest breaks to reduce health risks associated with irregular schedules and heavy workloads, while several member states have enacted laws to improve the conditions for delivery and platform workers (European Commission, 2021). In New Zealand, government–private sector collaboration has produced comprehensive safety and welfare guidelines, along with support programs to ease the impact of prolonged and irregular shifts (Ministry of Business, 2018). The Government of Canada is also exploring reforms to better protect digital platform workers (Employment and Social Development Canada, 2024). These findings suggest that the MFDWs’ health challenges are closely linked to the structural characteristics of their work environments, highlighting the need for a comprehensive strategy and international cooperation to build healthier workplaces (Employment and Social Development Canada, 2024; European Commission, 2021; Ministry of Business, 2018).

The findings indicate that MFDWs generally adopt a passive approach to health management, primarily due to the imbalance between high job demands and insufficient resources. According to the Job Demands–Resources (JD-R) model, prolonged working hours, physical strain, and income insecurity represent excessive job demands that deplete workers’ physical and psychological energy (Bakker & Demerouti, 2007). In contrast, the absence of institutional support, such as access to regular health checkups, social protection, or structured rest periods, reflects a lack of job resources that could otherwise mitigate these stressors. This imbalance leads to fatigue, unhealthy coping behaviours, and neglect of preventive health activities (Frank et al., 2023; Nguyen-Phuoc et al., 2023; Nilsen & Kongsvik, 2023; Taylor et al., 2023). From this perspective, the passive health management observed among Korean MFDWs is not merely an individual choice but a predictable outcome of systemic factors inherent in precarious MFDWs. Integrating the JD-R framework clarifies how excessive physical and economic demands, combined with limited resources, contribute to the deterioration of health behaviours and outcomes (Bakker & Demerouti, 2007). Therefore, interventions should focus on both reducing job demands and enhancing job resources, such as implementing mandatory rest policies, providing regular health screenings, and offering financial or institutional support to promote sustainable well-being among MFDWs (EU-OSHA, 2022; ILO, 2021).

### Implications for occupational health practice

This study revealed that MFDWs face significant challenges, including negligent driving, poor working conditions, unhealthy lifestyle habits, and a passive approach to health management. The strong correlation between delivery volume and income intensifies time pressure, subsequently leading to risky driving behaviours, irregular eating patterns, insufficient sleep, and increased substance use during work hours. Based on these findings, occupational health professionals should adopt multifaceted interventions addressing structural factors, such as implementing flexible scheduling, legally mandated break times, and regular distribution and inspection of essential safety equipment, as well as individual health-coping strategies. Providing on-site health consultations, personalised stress management counselling, and peer support groups is essential to adopt a more proactive approach to managing their health. These preliminary findings highlight areas for larger-scale research that could eventually inform evidence-based policy.

### Applying research to occupational health practice

Motorcycle-based food delivery workers (MFDWs), also known as platform workers, recognise traffic accidents as a critical health concern, but engage in risky driving behaviours to expedite deliveries and increase order acceptance. From the perspective of precarious work theory, these behaviours can be interpreted as rational adaptations to employment instability and income insecurity. The pressure to maximise earnings in a system without stable contracts or institutional protection compels workers to prioritise immediate financial survival over long-term health maintenance. This theoretical lens emphasises that MFDWs’ risky behaviours are not individual failings but structural outcomes of precarious gig work. These insights suggest that occupational health services should consider implementing tailored interventions that incorporate both health promotion and safety enhancement. Moreover, the findings indicate that improvements in delivery platform features and the integration of health support systems within occupational health practices may be important considerations for addressing the vulnerabilities associated with precarious employment.

### Strengths and limitations

This qualitative study provides valuable insights into the lived experiences and health perceptions of MFDWs in South Korea, a population that remains underrepresented in occupational health research. A key strength of this study lies in its in-depth interview design, which enabled a detailed exploration of MFDWs’ health behaviours, occupational risks, and coping strategies that are often overlooked in quantitative research. However, this study was conducted with a small sample of nine participants, all of whom were from a specific geographic area. Moreover, as the number of female MFDWs is relatively small compared to males, recruitment was challenging; thus, only male MFDWs were included in this study. Future large-scale studies involving both male and female MFDWs from diverse regions are recommended to generate more substantial and more generalisable evidence.

## Supplementary Material

Appendix_1_251201.docx Appendix_1_251201.docx

## Data Availability

The data presented in this study are available upon reasonable request from the corresponding author.

## References

[cit0001] Acs, Z. J., Song, A. K., Szerb, L., Audretsch, D. B., & Komlósi, É. (2021). The evolution of the global digital platform economy: 1971–2021. *Small Business Economics*, *57*(4), 1629–1659. 10.1007/s11187-021-00561-x

[cit0002] Antonucci, L. (2025). The lived experiences of the welfare state of platform workers: The barriers to accessing social protection in Italy, Sweden and the United Kingdom. *International Journal of Social Welfare*, *34*(1), e12708. 10.1111/ijsw.12708

[cit0003] Bakker, A. B., & Demerouti, E. (2007). The job demands–resources model: State of the art. *Journal of Managerial Psychology*, *22*(3), 309–328. 10.1108/02683940710733115

[cit0004] Boniardi, L., Campo, L., Prudenzi, S., Fasano, L., Natale, P., Consonni, D., Carugno, M., Pesatori, A. C., & Fustinoni, S. (2024). Occupational safety and health of riders working for digital food delivery platforms in the city of Milan, Italy. *La Medicina del Lavoro*, *115*(5), e2024035. 10.23749/mdl.v115i5.16278 39450630 PMC11562669

[cit0005] Braun, V., & Clarke, V. (2006). Using thematic analysis in psychology. *Qualitative Research in Psychology*, *3*(2), 77–101. 10.1191/1478088706qp063oa

[cit0006] Cheng, Y., Cheng, W. -J., Lin, R. -T., Wang, Y. -T., & Ko, J. -J. R. (2024). Associations between labor control through digital platforms and workers’ mental well-being: A survey of location-based platform workers in Taiwan. *Safety and Health at Work*, *15*(4), 419–426. 10.1016/j.shaw.2024.08.003 39697321 PMC11650788

[cit0007] Creswell, J. W., & Poth, C. N. (2016). *Qualitative inquiry and research design: Choosing among five approaches* (4th ed.). SAGE Publications.

[cit0008] Employment and Social Development Canada. (2024 June 21). *Legislative changes to support federally regulated employees*. Government of Canada. https://www.canada.ca/en/employment-social-development/news/2024/06/legislative-changes-to-support-federally-regulated-employees.html

[cit0009] European Agency for Safety and Health at Work. (2022). *Digital platform work and occupational safety and health: Overview of regulation, policies, practices and research*. Publications Office of the European Union. https://osha.europa.eu/en/publications/digital-platform-work-and-occupational-safety-and-health-overview-regulation-policies-practices-and-research

[cit0010] European Commission. (2021December 9). **Commission proposals to improve the working conditions of people working through digital labor platforms* [Press release]*. https://ec.europa.eu/commission/presscorner/detail/en/ip_21_6605

[cit0011] Frank, J., Mustard, C., Smith, P., Siddiqi, A., Cheng, Y., Burdorf, A., & Rugulies, R. (2023). Work as a social determinant of health in high-income countries: past, present, and future. *Lancet*, *402*(10410), 1357–1367. 10.1016/s0140-6736(23)00871-1 37838441

[cit0012] Goel, V., Unny, B. R., Shome, S., & Gupta, Y. (2024). Digital labor: A systematic literature review and bibliometric analysis. *International Journal of Organizational Analysis*, *32*(5), 967–1007. 10.1108/IJOA-12-2022-3558

[cit0013] Hajiheydari, N., & Delgosha, M. S. (2024). Investigating engagement and burnout of gig-workers in the age of algorithms: An empirical study in digital labor platforms. *Information Technology & People*, *37*(7), 2489–2522. 10.1108/ITP-11-2022-0873

[cit0014] Holloway, I., & Galvin, K. (2017). *Qualitative research in nursing and healthcare* (pp. 143–147 4th ed.). John Wiley & Sons.

[cit0015] International Labor Organization. (2021). *World employment and social outlook 2021: The role of digital labour platforms in transforming the world of work*. International Labor Organization.

[cit0016] Lee, S. Y., & Park, J. T. (2022). Analysis on factors contributing to motorcycle accidents of food delivery riders. *Journal of the Korean Society of Safety*, *37*(1), 70–77. 10.14346/JKOSOS.2022.37.1.70

[cit0017] Mbare, B. (2023). Psychosocial work environment and mental well-being of food delivery platform workers in Helsinki, Finland: A qualitative study. *International Journal of Qualitative Studies on Health and Well-Being*, *18*(1. 2173336. 10.1080/17482631.2023.2173336 36730307 PMC9897739

[cit0018] Ministry of Business, Innovation and Employment, New Zealand. (2018). **Health and safety at work strategy 2018–2028: Consultation document*.* Ministry of Business, Innovation and Employment. https://www.mbie.govt.nz/dmsdocument/4305-health-and-safety-at-work-consultation-document

[cit0019] Ministry of Employment and Labor, Republic of Korea, Division of Occupational Safety Standards. (2021December 27). Announcement of inspection results on food delivery platform workplaces. [Press release] https://www.moel.go.kr/news/enews/report/enewsView.do?news_seq=13100

[cit0020] Moon, J. -H., & Kim, H.-K. (2023). A study on the improvement of safety management for delivery platform workers. *Journal of the Society of Disaster Information*, *19*(1), 84–96. 10.15683/KOSDI.2023.3.31.084

[cit0021] Moon, B., Lee, S., & Jung, K. (2022). Characteristic analysis of occupational motorcycle accidents for food delivery workers by employment status. *Journal of the Korean Society of Safety*, *37*(6), 118–127. 10.14346/JKOSOS.2022.37.6.118

[cit0022] Nguyen, M. H., Nguyen-Phuoc, D. Q., Nguyen, N. A. N., & Oviedo-Trespalacios, O. (2024). Distracted on duty: A theory-based exploration of influences leading to mobile phone distracted riding among food delivery workers. *Accident Analysis & Prevention*, *202*, 107538. 10.1016/j.aap.2024.107538 38703589

[cit0023] Nguyen-Phuoc, D. Q., Ngoc Thi Nguyen, L., Ngoc Su, D., Nguyen, M. H., & Oviedo-Trespalacios, O. (2023). Deadly meals: The influence of personal and job factors on burnout and risky riding behaviors of food delivery motorcyclists. *Safety Science*, *159*, 106007. 10.1016/j.ssci.2022.106007

[cit0024] Nilsen, M., & Kongsvik, T. (2023). Health, safety, and well-being in platform-mediated work – A job demands and resources perspective. *Safety Science*, *163*, 106130. 10.1016/j.ssci.2023.106130

[cit0025] Papakostopoulos, V., & Nathanael, D. (2021). The complex interrelationship of work-related factors underlying risky driving behavior of food delivery riders in Athens, Greece. *Safety and Health at Work*, *12*(2), 147–153. 10.1016/j.shaw.2020.10.006 34178391 PMC8209359

[cit0026] Popan, C. (2024). Embodied precariat and digital control in the “Gig Economy”: The mobile labor of food delivery workers. *Journal of Urban Technology*, *31*(1), 109–128. 10.1080/10630732.2021.2001714

[cit0027] Sun, G. (2023). Quantitative analysis of online labor platforms’ algorithmic management influence on psychological health of workers. *International Journal of Environmental Research and Public Health*, *20*(5), 4519. 10.3390/ijerph20054519 36901527 PMC10002000

[cit0028] Taylor, K., Van Dijk, P., Newnam, S., & Sheppard, D. (2023). Physical and psychological hazards in the gig economy system: A systematic review. *Safety Science*, *166*, 106234. 10.1016/j.ssci.2023.106234

[cit0029] Vignola, E. F., Baron, S., Abreu Plasencia, E., Hussein, M., & Cohen, N. (2023). Workers’ health under algorithmic management: Emerging findings and urgent research questions. *International Journal of Environmental Research and Public Health*, *20*(2), 1239. 10.3390/ijerph20021239 36673989 PMC9859016

[cit0030] Xu, Y., & Liu, D. (2021). Decent work for the digital platform workers. A preliminary survey in Beijing. *Digital Law Journal*, *2*(1), 48–63. 10.38044/2686-9136-2021-2-1-48-63

[cit0031] Yu, D., Zhang, J., & Yun, G. (2024). Delivery riders’ safety and delivery efficiency in on-demand food delivery industry: The moderating role of monitoring algorithms. *Research in Transportation Business & Management*, *55*, 101143. 10.1016/j.rtbm.2024.101143

