# Supplementary material for: Health and health management among motorcycle-based food delivery workers in South Korea: a qualitative interview study
Source: Int J Qual Stud Health Well-being. 2026 Jan 11;21(1):2613971. doi: 10.1080/17482631.2026.2613971 (PMC12794690; doi:10.1080/17482631.2026.2613971)
Supplement: Appendix_1_251201.docx [file ZQHW_A_2613971_SM1411.docx]

**Interview Guide**

Hello, thank you for participating in this interview about the health status and health management experiences of motorcycle-based food delivery workers.
There are no right or wrong answers—please feel free to share your personal and indirect experiences, as well as your thoughts and opinions.
I will ask some questions, and you may respond freely. The interview will be audio-recorded and later transcribed for analysis. Your name or any identifiable information will not appear anywhere in the study, so please do not worry about confidentiality.

| Opening Question | - How would you describe the health of motorcycle-based food delivery workers? |
| --- | --- |
| Main Question 1 | - Could you describe your current health status while performing delivery work?  If it is difficult to answer, the interviewer may provide the examples below for clarification. (e.g., fatigue, sleep, digestion, hearing, musculoskeletal symptoms) |
| Main Question 2 | - Please tell us about your health behaviors while working as a motorcycle-based food delivery worker.  If it is difficult to answer, the interviewer may provide the examples below for clarification.  (e.g., eating habits, physical activity, alcohol consumption, smoking) - How do you usually manage your health? |
| Main Question 3 | - What kinds of discomfort or difficulties do you experience due to the surrounding environment while making deliveries?  If it is difficult to answer, the interviewer may provide the examples below for clarification.  (e.g., vibration from the motorcycle, road noise during day and night, air quality such as fine dust, glare or light reflection, heat or cold)  - Have you ever experienced verbal abuse, insults, or threats during work? If so, could you describe those experiences in detail? |
| Main Question 4 | - How do you usually cope with or relieve stress arising from your delivery work? |
| Closing Question | 1. I’ve summarized what you said. Does this sound accurate? Please let me know if anything is incorrect or not what you meant. 2. Is there anything else you would like to add or emphasize that we haven’t discussed? |
